# The Role of Counter-Ions in Peptides—An Overview

**DOI:** 10.3390/ph13120442

**Published:** 2020-12-03

**Authors:** Karol Sikora, Maciej Jaśkiewicz, Damian Neubauer, Dorian Migoń, Wojciech Kamysz

**Affiliations:** Department of Inorganic Chemistry, Faculty of Pharmacy, Medical University of Gdańsk, 80-210 Gdańsk, Poland; maciej.jaskiewicz@gumed.edu.pl (M.J.); damian.neubauer@gumed.edu.pl (D.N.); dorianmig@gumed.edu.pl (D.M.); wojciech.kamysz@gumed.edu.pl (W.K.)

**Keywords:** peptides, counter-ions, drug formulation, analytical techniques, physicochemical, counter-ion exchange, peptide drugs, peptide synthesis

## Abstract

Peptides and proteins constitute a large group of molecules that play multiple functions in living organisms. In conjunction with their important role in biological processes and advances in chemical approaches of synthesis, the interest in peptide-based drugs is still growing. As the side chains of amino acids can be basic, acidic, or neutral, the peptide drugs often occur in the form of salts with different counter-ions. This review focuses on the role of counter-ions in peptides. To date, over 60 peptide-based drugs have been approved by the FDA. Based on their area of application, biological activity, and results of preliminary tests they are characterized by different counter-ions. Moreover, the impact of counter-ions on structure, physicochemical properties, and drug formulation is analyzed. Additionally, the application of salts as mobile phase additives in chromatographic analyses and analytical techniques is highlighted.

## 1. Introduction

**Figure 1:**
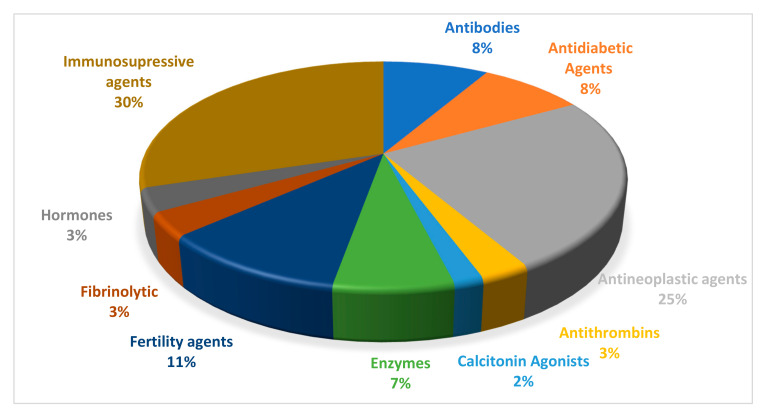
Application of FDA-approved peptide drugs.

Peptides along with proteins constitute the largest group of mediators of cellular processes making them pivotal for the functioning of many organisms. For this reason, peptides are considered as attractive patterns for the development of new drugs and therapeutic routes [1,2]. Despite the fact, that the breakthrough of peptide synthesis dates back to the 1960s (when the solid-phase synthesis was developed by Merrifield), peptides are still considered as poor drug candidates, mostly because of their poor bioavailability and the tendency to be metabolized before reaching the site of action. Moreover, their synthesis is expensive and time-consuming, they are chemically unstable (pH and thermal degradation, oxidation), and difficult to maintain. Nevertheless, some synthetic strategies and recent developments provide an opportunity to overcome these limitations [3]. Additionally, some approaches allow to modulate the pharmacokinetics and enhance their specificity [4]. For this reason, more and more synthetic peptides and peptide-based drugs are reaching the market. However, what are the main therapeutic areas for these compounds? The majority of marketed peptides are homologues of hormones or analogues simulating their action. Primarily, they were isolated from natural sources. The first was insulin. This hormone was isolated from the canine and bovine pancreas and introduced into the clinic in the 1920s for treatment of diabetes mellitus [5]. Nearly 30 years later, adrenocorticotrophic hormone (ACTH) was isolated from livestock pituitary glands and introduced to treat a variety of endocrine disorders [5]. At some time, an enormous progress in chemical sciences resulted in sequence elucidation and chemical synthesis of peptides. In 1953, oxytocin was the first therapeutic peptide obtained by chemical synthesis [6]. This nonapeptide, produced by hypothalamus is usually applied to initiate or strengthen contractions of uterus when the termination of pregnancy is medically indicated. Since that time, rapid progress in synthetic techniques resulted in several bioactive peptides, such as vasopressin (1962), insulin (1965), enkephalin (the 1970s), leuprorelin (1984), or octreotide (1988) [4]. During the past decades over 60 therapeutic peptides were approved by authorities and commercialized (Figure 1). The global market for peptide-based drugs was estimated to exceed USD 70 billion in 2019 [7].

This article discusses the role of counter-ions in peptides including drug formulation, the influence on physico-chemical properties and methods of counter-ion exchange, identification, and quantification.

## 2. Influence of Counter-Ions in Peptides on the Physico-Chemical Properties

### 2.1. Counter-Ion Impact on Peptides Structure

**Figure 2:**
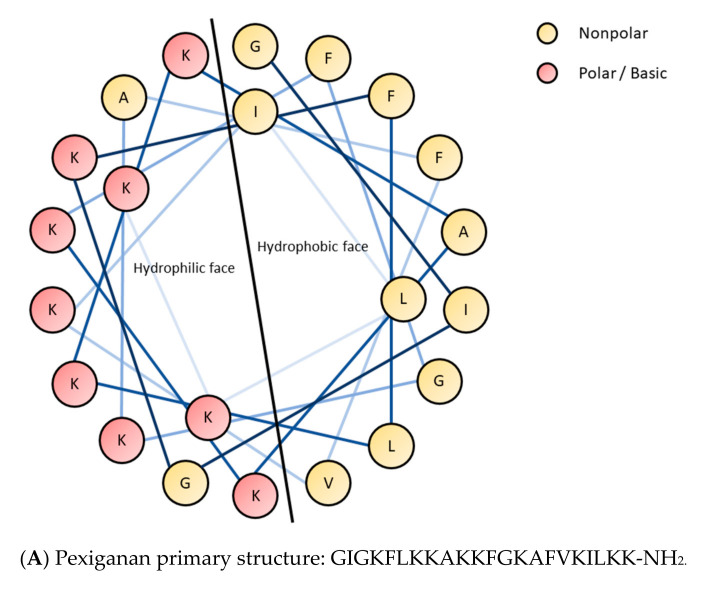

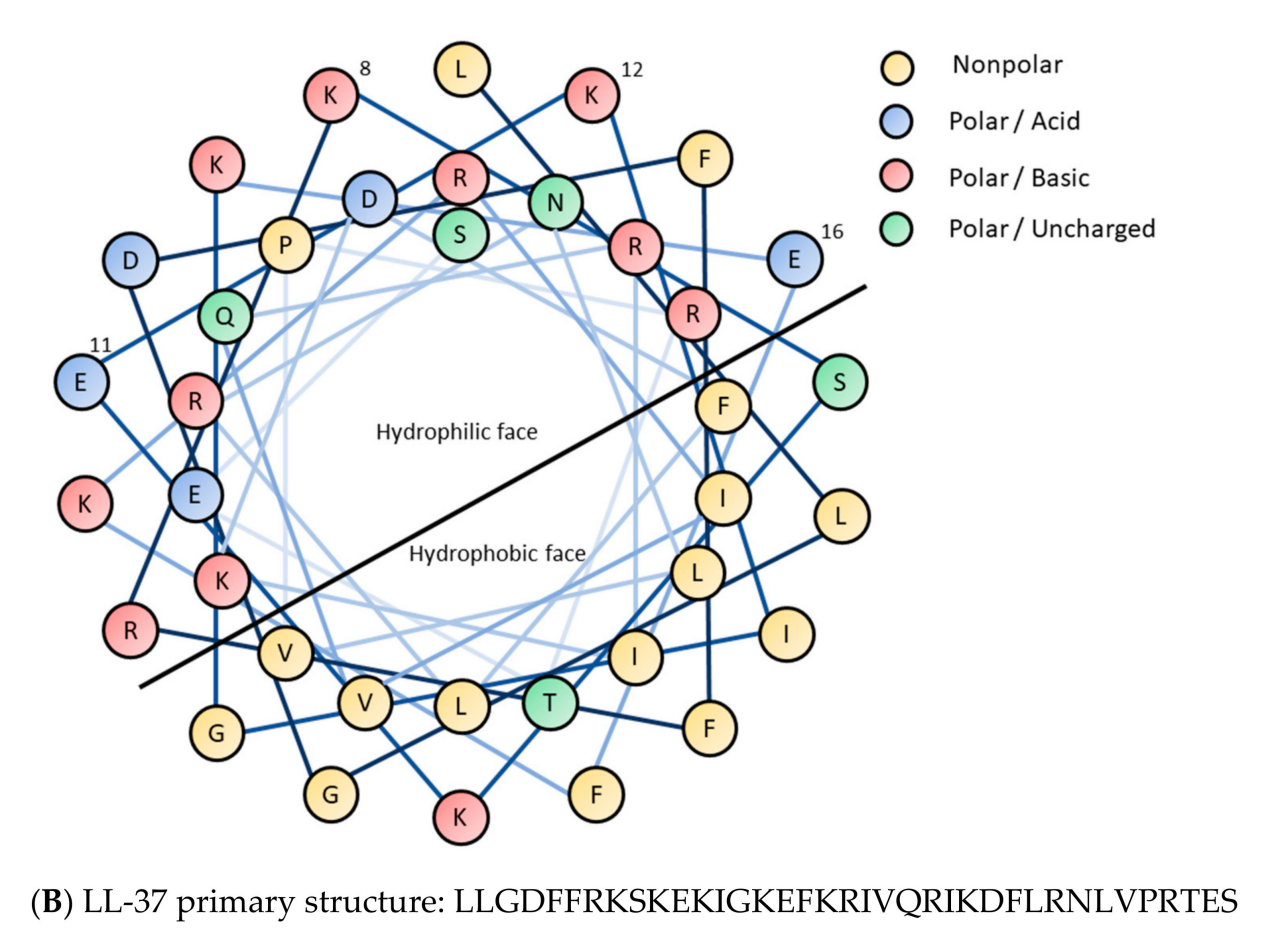
Helical-wheel projections of pexiganan (**A**) and LL-37 (**B**) [12] Blue lines represent peptide bonds and black line divide molecule into hydrophobic and hydrophilic face.

Peptides interact with charged molecules mainly due to their basic and acidic amino acid residues or lone pairs of oxygen, nitrogen, and sulphur atoms. These interactions can be classified as charge-to-charge or lone pair-to-cation ones. Here, we focus on the counter-ion effect on biological activities, physico-chemical properties, and final therapeutic formulation, of peptides but nonionic species will not be discussed in detail. As a matter of fact, charge-to-charge interactions are associated with a side-chain of the peptide and the type of counter-ion, of the interacting molecule. This can be either an anion (TFA^−^, AcO^−^, Cl^−^, etc.) or a cation (Na^+^, K^+^, Ca^2+^, etc.) In peptides, side-chains of aspartic acid (Asp, D), glutamic acid (Glu, E), and C-terminal carboxyl groups are anionic. Again, basic amino acid residues are in histidine (His, H), lysine (Lys, K), arginine (Arg, R), and *N*-terminal amino group. Additionally, more charged amino acid residues can be found for example those nonproteinogenic ones in γ-carboxyglutamic acid and ornithine. Furthermore, side chains of cysteine (Cys, C) and tyrosine (Tyr, Y) are able to dissociate, but their pK_a_ values are relatively high (8.14 and 10.10, respectively) [8] so at a physiological pH they are mostly protonated. The basicity and acidity (pK_a_), as well as interactions with counter-ions, generally depend on the environment. Dehydration that occurs in a hydrophobic protein interior can affect pK_a_ values of acids and bases. Dissociation of carboxylic acids can be written as R-COOH⇆R-COO^−^ + H^+^; while equation for bases is R-NH_3_^+^⇆R-NH_2_ + H^+^. In general, lowering of the dielectric constant favors the neutral form of an ionizable group (Born effect). When comparing water and a dehydrated environment (lower dielectric constant) it can be stated that the neutral form is favored in the latter and therefore the pK_a_ of bases decreases unlike that of acids [9]. Specifically, pK_a_ values of lysine in pure water at 25 °C (dielectric constant, ε = 78.54) are 11.08, 9.05 (amino groups), and 1.76 (carboxyl group), while in a 70% ethanol (ε = 53.44) are 9.64, 8.48, and 2.19, respectively [10,11]. Moreover, a positively charged environment diminishes the pK_a_ of both acids and bases, while a negatively charged environment results in an opposite effect. Hydrogen bonds can also contribute to enhancing the pK_a_ when the hydrogen bonding is shifted to the protonated form and decreasing when a deprotonated form participates in the bonding [9]. Undoubtedly, counter-ions have an impact on peptides secondary structure. Usually, peptides tend to adopt a specific secondary structure to properly interact with their molecular targets and to evoke desired biological effects. For instance, such interactions are noticed in the case of antimicrobial peptides (AMPs), which are frequently α-helical, amphipathic, and they disrupt target cell membranes of microorganisms. In general, effective interactions with a membrane bilayer depend on the peptide characteristics such as well-defined hydrophobic and hydrophilic faces (usually positively charged). As a result, one side is engaged in interplay with lipid tails while another attracts a negatively charged polar head of membrane phospholipids. The maintenance of appropriate secondary structure also facilitates those interactions that improve the separation of two opposite regions. This fact can be explained by means of helical-wheel projections of the α-helical antimicrobial peptides (human cathelicidin—LL-37, and pexiganan—MSI-78) presented in Figure 2.

**Figure 3:**
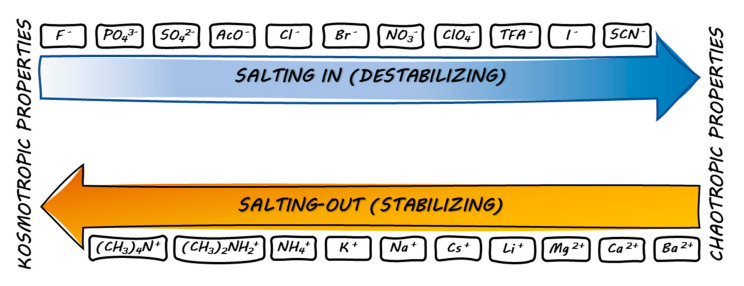
Kosmotropic and chaotropic properties of cations and anions [33,34].

Both peptides adopt an α-helical conformation where the polar amino acid residues are mostly on one side of the helix and those nonpolar on the other. Therefore, if the α-helical structure (or any other structure) provides an effective charge distribution favorable for antimicrobial or any other biological activity, then any divergence from the optimal secondary structure will lead to modifying the specific biological effects. Counter-ions that occur in peptides environment can also affect their secondary structure and consequently their biological properties. For example, Johanson et al. [13] investigated those interactions for LL-37. The effect of different counter-ions was found to be different. Among the tested anions (Cl^−^, HCO_3_^−^, SO_4_^2−^, and TFA^−^) chloride was the least efficient in induction of the helical structure. In fact, the secondary structures of LL-37 and pexiganan are more complex. In dodecylphosphocholine (DPC) LL-37 shows a break between two helical fragments (helix-break-helix) stabilized by a salt bridge (hydrogen-bonded ion pair) between Lys12 and Glu16 (Figure 2). Moreover, interactions between Lys8 and Glu11 (Figure 2) are also probable owing to their proximity [14]. In this case, counter-ions are not provided by the environment but result from ionic fragments of peptides in the side chains, a zwitterionic form. Those specific interactions between the Asp/Glu and Arg/Lys (i, i + 3/4) side-chains are due to the presence of such issues as electrostatic, hydrophobic, hydrogen bonding, and conformational preferences which depend on the pH and the length of the side chain [15,16,17]. In pexiganan, salt bridges are missing and its molecules self-associate through phenylalanine zipper to form dimers in DPC [18]. Another example of counter-ion impact on the peptide secondary structure has been reported by Gaussier et al. In their research on Pediocin PA-1, the impact of TFA^−^ was analyzed and a slight increase in helical structures as compared to that of Cl^−^ was noticed. However, no significant difference in antimicrobial activity was found [19]. On the other hand, anion binding can also be intentionally achieved through cyclic peptides. Usually, these artificial receptors are cyclic hexapeptides and their modifications—cyclopeptido-mimetics, biscyclopeptides (two subunits) with a linker, and solubilizing groups that enhance solubility in water. In addition, linear peptides with modified *C*- and *N*-terminal were also investigated. These molecular probes were designed for selective binding of anions such as F^−^, Cl^−^, Br^−^, I^−^, AcO^−^, SO_4_^2−^, NO_3_^−^, and H_2_PO_4_^−^ [20,21,22,23,24]. Furthermore, another aspect that relies on this phenomenon is the extraction of ions from the solution to another nonmiscible phase [25]. Often, these cyclic peptides (and molecules with peptide scaffolds) have elements of symmetry (axes of symmetry) and constrained conformations [26]. Anions are bonded through hydrogen bonds with NH peptide groups. Typically, backbone hydrogen bond donors (NH groups) are pointing into the peptide internal cavity to form hydrogen bonds with the anion (1:1 complex). In effect, the complex has a flattened conformation [20,25]. Besides, sandwich complexes with two cyclopeptide molecules and one anion are also under investigation (2:1 complex). In these complexes, the anion is desolvated and entrapped between two peptide rings [21,27]. On the other hand, peptides can also interact with cations occurring in the environment through acidic groups such as carboxylic ones, phosphates (phosphorylated serine, threonine, and tyrosine), lone electron pairs, and aromatic rings (cation-π interactions). For example, β-casein (1–25) whose primary structure is RELEELNVPGEIVE(*p*S)L(*p*S)(*p*S)(pS)EESITR (*p*S—phosphoserine) has a number of acidic residues that can easily bind cations. Moreover, this peptide is characterized by anticariogenic properties as it binds to amorphous calcium phosphate at the tooth surface. As a result, teeth are remineralized and protected from demineralization. Furthermore, it has been demonstrated that the secondary structure of β-casein (1–25) strongly depends on the counter-ion type. For example, monovalent cations (Na^+^ and NH_4_^+^) have no impact on the structure and therefore this peptide is conformationally unordered and flexible, but a divalent cation, Ca^2+^, interacts with peptide charged groups and, in effect, the β-casein fragment is ordered in one loop-type conformation and three β-turn regions [28]. Another intriguing example of a peptide that binds calcium ions is Laspartomycin C that exhibits antimicrobial activity against Gram-positive bacteria and similar to daptomycin (FDA approved cyclic lipopeptide) is classified as a calcium-dependent antibiotic (CDA). Moreover, the mode of action of this lipopeptide relies on binding to undecaprenyl-phosphate (C_55_-P) associated with inhibition of essential cell-wall precursor lipid II [29]. Furthermore, it has been shown that two calcium ions are essential for secondary structure stabilization and interaction with a phosphate group of C_55_-P [30]. It should be emphasized that the high-resolution crystal structure showed that Ca^2+^ ions are coordinated to the lone pairs of backbone carbonyls, carboxyl groups in the side chains of aspartic acid, C_55_-P, and water molecules. Presumably, Laspartomycin C forms dimers in solution with some participation of calcium ions [31]. These few examples demonstrate specific interactions between ions and peptides and peptidomimetics. A general statement on protein and peptide interactions with ions has been offered by the Hofmeister series (lyotropic) of cations and anions. It was published by Hofmeister in the late 1900s and it classifies ions based on their ability to salt out macromolecules, i.e., proteins. Ions in each series are ordered in terms of increasing impact on peptide/protein solubility and structure. These effects were found to be more substantial for anions than for cations. Ions can be classified as kosmotropic when they stabilize the native state of macromolecules in water (salting-out) and chaotropic when they destabilize (salting-in) them (Figure 3). Chaotropes, as opposed to kosmotropes, do not favor interactions between water molecules (disrupt hydrogen bonding), and simultaneous destabilization of peptide/protein intramolecular interactions leads to enhanced solubility of nonpolar solutes and the loss of the native structure [32].

Anionic kosmotropes have a higher charge density and a stronger electric field than anionic chaotropes. In effect, a hydration layer is tighter and the most stabilizing anions (SO_4_^2−^, PO_4_^3−^) are also those which are the most hydrated. However, the analogous series of cations exhibited an opposite relation [33,34]. Importantly, this classification into chaotropes and kosmotropes has led to a great simplification. In fact, in biomolecules this issue includes ion hydration resulting in different binding affinities between the ion and peptide backbone, negatively or positively charged side chains. It has been stated that salting-out relies on competition between salt ions and peptide/protein charged/polarized groups for interactions with solvent molecules (typically water). Moreover, cations and anions interact with ionized groups of peptide molecule and polarized peptide bonds, or even Cα [35]. As long as repulsive forces between macromolecules prevail, no precipitation will be noticed. In fact, the minimum solubility of peptides and proteins is noticed at their isoelectric point (pI) owing to the high tendency to attract each other. At the isoelectric point, the molecule has no net electrical charge. In effect, at pI, electrostatic repulsions between molecules are minimal. Moreover, when interactions with salts dominate, macromolecules start to aggregate and precipitate, which strongly relies on hydrophobic interactions. In fact, the minimal concentration of salts when precipitation occurs depends on the solvent, particular salt (cation and anion), precipitated peptide/protein, pH, and temperature [36,37]. An ideal salting-out can be described as precipitation of macromolecules with no denaturation. Simultaneously, the surface tension increases, and the solubility of hydrocarbons diminishes. Furthermore, the risk of denaturation and destabilization of macromolecule increases when the salt is more chaotropic. This is one of the reasons why ammonium sulphate, as a kosmotropic salt, is commonly used for salting-out of proteins. Both, the ammonium (NH_4_^+^) and sulphate (SO_4_^2−^) ions are highly kosmotropic. Recent studies have shown that the efficiency in salting-out depends not only on the anion or cation type but also on groups with which particular ions might interact. When an anion interacts with the peptide backbone normal anionic Hofmeister ordering takes place where weakly hydrated anion interaction is stronger and is less efficient in salting-out. Interestingly, anions interacting with positively charged side chains follow the reversed Hofmeister series—strongly hydrated anions interact more strongly than less hydrated ones. Again, cations follow standard Hofmeister ordering where poorly hydrated cations interact more weakly and are more efficient in salting-out [32,38]. In general, it can be said that salting-out relies mainly on ionic interactions whereas the backbone is irrelevant. On the other hand, it depends on the number of charged groups that might be engaged in interaction at a particular pH. Interestingly, l-proline or l-arginine salts added to a protein solution can act as suppressors of aggregation [39,40]. l-arginine counter-ion is commonly used as a potent aggregation suppressor. Briefly, it may enhance the diffusional barrier between macromolecules through the surrounding network of bridged ions, increase the thermodynamic stability of the native state of the protein, and increase the medium viscosity. The ability of l-arginine salt to inhibit aggregation of α-chymotrypsinogen declines consistently with Hofmeister series—l-ArgH(H_2_PO_4_) is the most promising suppressor while l-ArgHSCN is the least [41]. Furthermore, some peptides are also prone to aggregate by forming fibrils. For instance, the most prominent example of such structures is β-amyloid (Aβ) being an amphiphilic peptide (36–43 amino acids). Aβ molecules can easily form either dimeric and tetrameric quaternary structure or amyloid aggregates (fibrils, amyloid plaques). It should be noted that amyloid fibrils as plaques found in the brain play a crucial role in the development of Alzheimer’s disease. It has been found that counter-ions strongly influence the kinetics of fibril formation. These processes are also accelerated when Cl^−^ is a counter-ion, in contrast to TFA salt. Moreover, subsequent formation of an α-helical intermediate, β-structures, and fibrils from unordered Aβ were observed only with TFA^−^ counter-ion, while Cl^−^ Aβ undergoes the transformation from unordered structure to fibrils directly via β-sheet conformation [42,43,44]. Interestingly, it has been shown that the counter-ion type can have an effect on the thermal stability of peptide nanofibers [45]. In general, amphiphilic peptides are prone to self-assembly and form different structures, such as helical ribbons, micelles, nanofibers, nanotapes, nanotubes, oligomers, and twisted tapes. Peptide environment (solvent), temperature, concentration, pH, enzymes, cosolved molecules, and ions have a great impact on the self-assembly process [46,47,48].

### 2.2. Counter-Ions in High-Performance Liquid Chromatography (HPLC) Analysis and Purification of Peptides

Initially, peptides and proteins were isolated from natural sources, including tissues or cell cultures cultivated in bioreactors. Although, purification of compounds from these complex mixtures with plenty of similar compounds present in the matrix is a challenging task even for experienced scientists. However, not only isolation from natural sources can be problematic. The same situation can be observed after chemical synthesis, where desired substances are part of a mixture containing unreacted substrates, catalysts, solvents, and by-products. Over the decades, multiple techniques were invented for effective isolation of compounds such as salting-out (just mentioned); precipitation with salts, organic solvents; pH manipulation; extraction; solid-phase extraction; adsorptive chromatography; ion-exchange chromatography; electrophoresis; size-exclusion chromatography; affinity chromatography; hydrophilic-interaction chromatography and liquid chromatography. To do this, systems including normal phase (NP-HPLC) or reversed phase (RP-HPLC) and others are used of which RP-HPLC is routinely applied for analysis and purification of peptides and small proteins. In this technique, the stationary phase is usually composed of silica modified with C18 alkyl chains, less frequently with C8, C4, and others. Mobile phase typically consists of water and acetonitrile (methanol or ethanol) with additives—buffers or acids alone. To prevent crystallization and clogging of HPLC elements and column, mobile phase additives have to be soluble in both organic solvents and water and also in their mixtures in any proportion. Usually, to improve the solubility of the analyzed macromolecules (peptides, proteins) pH of the solution should differ from the pI. Low pH maintains both acidic and basic residues in the protonated form. Hence the charged species interact with counter-ions derived from deprotonated acid molecules (charge shielding), while free silanol groups of the stationary phase undergo protonation (no charge). Moreover, acidic additives interact with positively charged peptide groups (e.g., *N*-terminal amino group, Arg, His, Lys) to form an ion-pair and thus affect molecule hydrophobicity. Indeed, each positive charge has an equal contribution to peptide retention unlike counter-ion type [49]. In effect, peptide ion–stationary phase ion interactions (secondary interactions) are substantially diminished, so the retention depends mainly on the hydrophobicity of the molecule. Trifluoroacetic (TFA), acetic (AcOH), formic (FA), phosphoric acid, and hydrochloric acids at a concentration of 0.1% (*v*/*v*) are the routinely used acidic additive of the mobile phase. Respective pKa values at 25 °C are 0.52, 4.76, 3.75, 2.16, 7.21, 12.32, and −7 [8,50]. All the acids just mentioned, except the phosphoric one, can be removed by lyophilization after peptide purification and therefore do not require the additional step of desalting. It is extremely important to maintain appropriate pH due to its substantial impact on the retention of charged molecules such as peptides [51]. Hence, the universal concentration of the acid added at 0.1% (*v*/*v*) is not always optimal and should be reconsidered before conducting the analysis. Sometimes a higher concentration (0.2–0.25%) offers a better resolution, reduces peak tailing [52], especially for those with multiple positive charges [53]. However, while increasing acid concentration, the risk of damaging HPLC column arises and it is essential to find a balance between separation and column stability. Some authors suggest applying calculation where TFA concentration in the mobile phase is equal to that of a peptide in the sample multiplied by the number of positive charges on the molecule, to achieve optimal resolution. However, it is emphasized that this cannot be applicable to peptides with high charge (above +3) [52]. Furthermore, the concentration of acid additive may have an impact on the conformation of proteins, which can be noticed as a change in their retention times in the RP-HPLC analysis. This effect is presumably based on stabilization of the molten-globule-like structure by ion-pairing, this furthermore facilitates entering the pores of the stationary phase and improves protein retention [54]. On the other hand, studies on albinterferon α-2b revealed that the acidic additive can induce protein dimerization through disulphide bridge formation and this phenomenon is concentration-dependent, but it occurs only when the protein is mildly oxidized [55]. It is noteworthy that proline as an uncharged residue can have a substantial impact on the peptide structure owing to its specific property, the tendency to *cis/trans* isomerization. It has been reported that addition of different salts in the solution of proline-based molecules can shift peptide bond to *cis* conformation, and moreover it is possible to maintain the new *cis/trans* ratio conformers over a wide temperature range [56]. One of the examples of the influence of proline residues on the results of RP-HPLC analyses is enalapril acting as an angiotensin-converting-enzyme (ACE) inhibitor. The isomerization leads to two isomers with different retention times which can be separated on RP-HPLC. Addition of a counter-ion modifier to the mobile phase can improve separation or even eliminate these bimodal peaks. Although the efficiency of separation depends on counter-ion type, also other conditions such as flow rate, temperature, pH, and mobile phase solvents are optimized. Counter-ion additives employed to enalapril analysis are, for example, cetyltrimethylammonium bromide (CTAB), sodium dodecyl sulphate (SDS), tetrabutylammonium hydrogen sulphate (TBAHS), and sodium 1-octanesulfonate (NaOS) [57,58]. In general, there are plenty of ion-pairing additives applicable in RP-HPLC analysis of peptides including acids i.e., TFA or phosphoric acids and NaCl, NaTFA, and NaClO4 salts [59]. Tarafder et al. [60] studied the influence of the type and level of additives to the mobile phase during the purification of peptides under overload column conditions. Interaction of the counter-ions (TFA and phosphates) from the mobile phase and formation of ion-pairs caused distinctly different chromatographic profiles including the change of peak shape, peak splitting, and even their disappearance. They noticed a competing ion-pairing effect between calcitonin and TFA and di-hydrogen phosphate. The phenomenon of peaks shape change was explained by counter-ions. One of the postulated mechanisms suggested that a peptide absorbed on the RP stationary phase acted as an ion-exchange molecule.

During analysis and identification of peptides, the HPLC systems are often coupled with electrospray ionization mass spectrometry (ESI-MS) detectors. In this case, some additives to the mobile-phase can affect ionization of the molecules and that is why they have to obey several criteria. First of all, they must be volatile (acid, base, or salt) for instance acetic acid, formic acid, ammonium acetate and formate, ammonium bicarbonate, ammonium hydroxide, triethylamine, or morpholine. Secondly, the ion pair formed between protonated peptide and counter-ion has to be easily separated during ionization at MS source. For this reason, one of the popular additives in RP-HPLC analyses of peptides, TFA, is not recommended for use in LC-MS, due to the relatively high ion suppression and lowering of sensitivity. Additionally, the loss of separation resolution is possible and should be considered. The first solution to this problem is to lower the concentration of TFA (from 0.05% to 0.01%) or replace it with another perfluorinated acid such pentafluoropropionic (PFPA), heptafluorobutyric (HFBA), or nonafluoropenatnoic acids (NFPA). The second one is to strike the balance between a mixture of additives i.e., acetic acid and low concentration of TFA [61]. In general, the increasing chain length of the perfluorinated acid (increasing its hydrophobicity) and its concentration will result in higher retention of positively charged peptides [62]. It is noteworthy that hydrophobicity of anionic ion-pairing agents increases in the order HCOOH ≈ H3PO4 < TFA < PFPA < HFBA < NFPA. Similarly, in hydrophilic interaction chromatography (HILIC) addition of anionic ion-pairing agents will reduce peptide retention with increasing hydrophobicity. Interestingly, cationic ion-pairing reagents such as diamylammonium (DAAA), dibutylamonium (DBAA), dihexylammonium (DHAA), and dipropylammonium acetates (DPAA) are likely to facilitate the change of retention time of glycopeptides with sialic acid residues in HILIC analysis [63].

## 3. Biological Activity of Peptides in the Form of Different Salts

### 3.1. Therapeutic Peptides

The role of counter-ions in pharmaceutical systems, especially in terms of the manufacturing processes or drug delivery is important. First of all, the total composition can affect the overall stability of the formulation. Moreover, excipient counter-ions paired with active pharmaceutical ingredients (APIs) can modulate drug efficacy, receptor engagement, cell penetration, and molecular conformation [64]. Therefore, the aspect of counter-ions has to be taken into account during the preparation of therapeutics. As previously stated, peptide synthesis is generally based on solid-phase procedures. The cleavage of the peptide and purification are performed using TFA. Thus, the cationic peptides are obtained as trifluoroacetate salts [65]. It is commonly known that TFA may interfere not only with physio-chemical characterization, but it also affects the in vivo experiments through influence on mammalian cells [66]. Sometimes it can be toxic and/or inhibits cell proliferation [67]. However, in some cases, it can act in the opposite way. For example, TFA was found to stimulate the growth of glioma cells in a dose-dependent manner or acts as an allosteric modulator of glycine receptors by increasing their activity at lower concentrations of glycine [68,69]. Furthermore, Ha et al. found that TFA activates the ATP sensitive potassium channels suggesting that it could mediate isoflurane-associated cardioprotection during myocardial ischemia and reperfusion [70,71]. On the other hand, Boullerne et al. investigated the impact of different counter-ions on the activity of MOG_35–55_ peptide (Myelin Oligodendrocyte Glycoprotein)—the popular immunogen applied for experimental induction of autoimmune encephalomyelitis [71]. They also found that the onset of the disease occurred approximately 5 days earlier when using MOG-B-containing trifluoroacetate as compared to that of peptide-containing acetates. However, most of the studies highlight the negative impact of TFA on the results of biological experiments. For instance, in a paper published by You et al. it was found that TFA-modified liver proteins are likely to induce T-cell responses and to enhance the production of proinflammatory cytokines [72,73]. Interestingly, trifluoroacetylated proteins were also found to elicit antibody responses [74]. For this reason, the TFA content is highly undesirable, especially for peptides intended for clinical and preclinical studies. Most of the peptide drugs and peptide-based therapeutics are provided as acetate salts (see Table 1). However, there are some peptides on the market that are formulated as trifluoroacetates. One of these is bivalirudin, a synthetic analogue of hirudin that has successfully been applied as a direct thrombin inhibitor during percutaneous coronary intervention [74]. Another one is corticorelin (also known as corticorelin ovine trifluate), a synthetic analogue of human peptide corticotropin-releasing factor (CRF) identical with ovine corticotropin-releasing hormone (oCRH) simulating the release of adrenocorticotropic hormone (ACTH). It is prescribed for use in differentiation of pituitary and ectopic production of ACTH in patients with ACTH-dependent Cushing’s syndrome. Moreover, several studies have indicated the ability of CRF to reduce the brain edema caused by brain tumors [74]. Interestingly, acetate of this compound in the form of the injectable drug (Xercept^®^) is currently running Phase III of clinical trials as an orphan drug in the treatment of peritumoral brain edema [75]. Although most of the applied counter-ions for peptides are acetates and chlorides, a second one is only rarely found among pharmaceuticals on the market. However, there is an exception. For example, Omiganan (MBI-226), an antimicrobial peptide that was studied in a series of clinical trials as hydrochloride [76]. With other peptides, especially, those characterized by a high molecular weight with very long amino acid chain, information about counter-ions is rather obscure. Therefore, these compounds are probably obtained either as recombinant peptides (rDNA) or the influence of counter-ions, in this case, seems to be marginal. In our previous article we studied the influence of counter-ion type on antistaphylococcal, and hemolytic activities as well as cytotoxicity against HaCaT cell line of several antimicrobial peptides (citropin, CAMEL, temporin A, pexiganan, and LL-37) [77]. Peptides with different counter-ions, acetates, chlorides, and trifluoroacetates, were prepared. All the tested compounds exhibited high activity against reference and clinical strains of *Staphylococcus aureus*. Moreover, some differences in biological activity between the tested salts were found. Among the peptides, the highest antistaphylococcal activity displayed CAMEL in the chloride form. Pexiganan acetate and temporin A trifluoroacetete exhibited the highest selectivity indexes. The results have shown that there is no simple correlation between counter-ion type and biological activity. Each case should be considered individually, and the type of counter-ion should be optimized each time to achieve optimal biological characteristic. Greber et al. investigated the influence of TFA counter-ion on antibacterial and antifungal activities of a series of short cationic lipopeptides. In most cases, removal of TFA increased antibacterial and antifungal activities, by lowering minimum inhibitory concentration (MIC) and minimum bactericidal concentration (MBC). In few, cases the presence of TFA anions brought a slight increase in activity against *K. pneumoniae* and *C. albicans*. The authors suggest that those observations can be partially explained by differences in molecular weights of peptides in zwitterion form and as salts [78].

### 3.2. Peptide Counter-Ions: Implications in Peptide Formulations

Counter-ions influence multiple peptide properties, their lipophilicity, self-assembly tendencies, and stability, among others. Hence, they have a significant impact on the peptide drug formulation profile. Up to date, most of the approved peptide pharmaceuticals are acetate salts (Table 1). There are three main reasons for this situation. Undoubtedly, the selection is associated with aforementioned trifluoroacetate toxicity. Another aspect is of historical nature—acetate counter-ions, used especially in early peptide drugs, were an outcome of countercurrent distribution (CCD) implementation as a last stage of purification process. Briefly, CCD is a multistage liquid–liquid extraction technique commonly used for biomolecule purification before introduction of high-resolution chromatography. In this technique, molecules are separated based on their partition coefficients specific for immiscible solvents used. Most of the systems designed for peptide purification included 1-butanol or 2-butanol as organic components along with aqueous solutions of acetic acid [82,83,84]. Counter-ion exchange to acetate is characterized by distinctly milder process conditions as compared to that with HCl (pH < 1), thus they theoretically ensure a safer process with a suppressed degradation propensity [65,66]. Moreover, salt screening implemented in peptide development might be beneficial for drug products in terms of their stability, safety, and patentability [85]. As an example, Beck et al. reported a superior stability a peptide HCl salt during stability studies as compared to that of TFA and acetate salts [86].

This section focuses on the role of counter-ion in peptide formulation. In view of unique characteristics of lipid-based nanocarrier systems utilizing peptides with hydrophobic counter-ions, recent advancements in this field are discussed in a separate subsection.

#### 3.2.1. Examples of Counter-Ion Influence on Peptide Formulation

Johnsson et al. in their patent application compared liquid crystalline formulations of octreotide acetate with those of the chloride salts. Both formulations were subjected to release and stability assays. Interestingly, those containing octreotide chloride exhibited approximately four times slower release rate as compared to that of the acetate formulation. Furthermore, the formulation based on octreotide chloride was characterized also by an increased stability. While only a slight drop in peptide concentration was found with octreotide chloride formulation stored for four weeks at 40 °C, an expressive decrease in peptide concentration took place in an octreotide acetate formulation stored under comparable conditions [87]. Similar, superior characteristics of the peptide chloride salts over those of the acetates were also reported by Yuhua et al. They reported that peptides with counter-ions of strong acids (e.g., hydrochloric acid) formulated into injectable biodegradable polymeric formulations exhibited suppressed tendency to interact with polymer molecules than those with counter-ions of weak acids (e.g., acetic acid) [88]. Cormier et al. found a suppressed tendency to fibrillation of a peptide in the presence of two different counter-ions (for example, acetate and chloride) as compared to single counter-ion. They explained this phenomenon as a result of fibrillation process disturbance due to nonuniformity of peptide units [89]. Another interesting influence of counter-ion on peptide formulation was reported by Deghenghi et al. who claimed that when trifluoroacetates or sulphates were used as counter-ions in Teverelix high concentration aqueous preparation, a microcrystalline, milky suspension was formed. In contrast, when Teverelix acetate was formulated in a similar manner, a gel with poor bioavailability in vivo was obtained. Such differences had a distinct impact on patient compliance, as far as the injection of such formulation for prolonged sustained delivery of peptide is considered [90]. Deasy et al. in their patent application presented the aforementioned hydrophobic ion pairing method for increasing transdermal bioavailability of peptide drugs [91]. Another patent application utilizing hydrophobic ion pairing as a means for increase peptide bioavailability was created by Botti et al. who discovered that a significant increase in oral mucosal delivery can be achieved when peptide drugs are paired with hydrophobic counter-ions, complexed with a crown compound and solubilized in a nonaqueous vehicle at a pH different from the isoelectric point of the peptide [92]. Further, Adjei et al. applied this method to improve leuprolide solubility in a propellant–water–ethanol cosolvent system, thus enabling pulmonary administration of the peptide with satisfactory efficiency (relative bioavailability ≈90%; dog model) [93]. Examples of counter-ion influence on peptide formulation are set out in Table 2.

#### 3.2.2. Peptide Hydrophobic Ion Pairing

**Figure 4:**
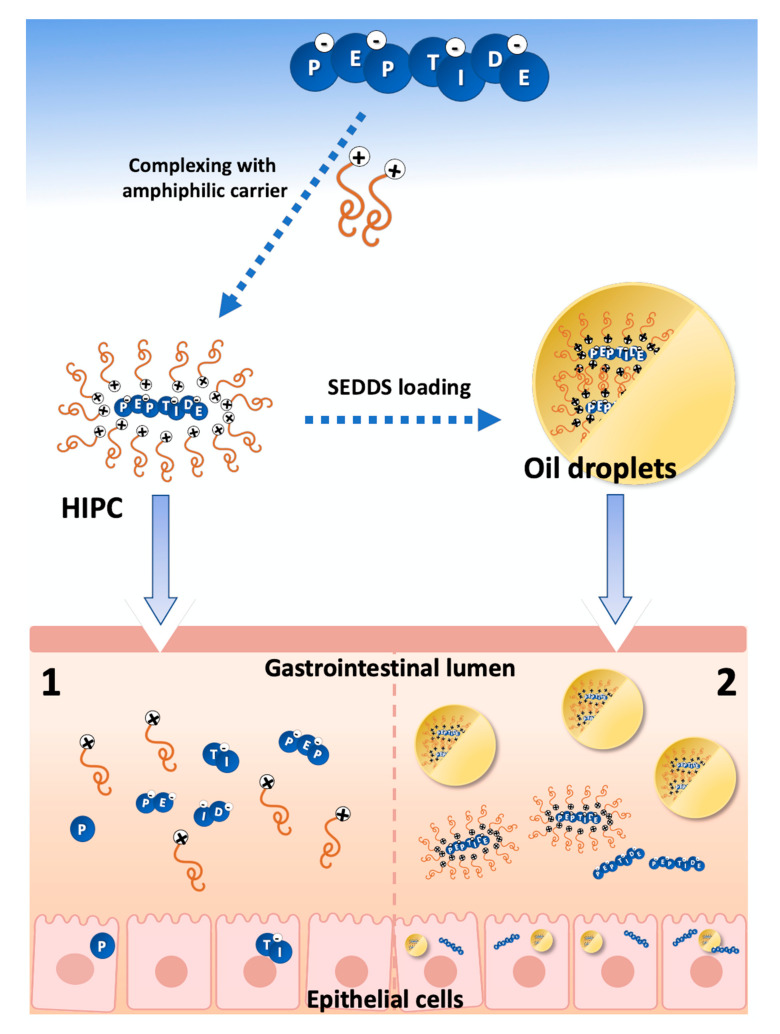
Self-emulsifying drug delivery systems (SEDDS) and hydrophobic ion pairing (HIP). 1—poor stability and degradation; 2—improved stability and enhanced delivery. Doted arrows—formation of HIP complex and SEEDS oil droplets. Arrows—adsorption in gastrointestinal lumen.

The use of most peptide drugs, based on hydrophilicity, and hence the poor membrane permeability, as well as low enzymatic stability, is restricted to intravenous, subcutaneous, and intramuscular administration. However, as those delivery routes are accompanied by pain and low patient compliance, several efforts were made to overcome administration limitations. One of the most promising tools for peptide drug delivery is lipid-based nanocarrier systems. Generally, a drug carrier can be defined as an umbrella term involving multiple vectors used in the process of drug delivery, such as liposomes, micelles, microspheres, nanoparticles, nanoemulsions, or self-emulsifying drug delivery systems (SEDDS). Since lipid-based nanocarriers can enhance both enzymatic stability and membrane permeability of molecules, they enable applicability of alternative delivery routes such as oral or pulmonary ones (Figure 4). On the other hand, because of the highly hydrophilic character of most peptides, their entrapment efficiency in lipid-based nanocarriers is poor. Up to date, several techniques conceived to increase lipophilicity have been developed and reported. One of these is hydrophobic ion pairing (HIP) (Figure 4), a process based on the pH-related, stoichiometric replacement of polar counter-ions with an ionic surfactant of identical charge, but of reduced hydrophilicity. Such complex (hydrophobic ion paired complex—HIPC) is characterized by a lower solubility in aqueous solutions, and an enhanced solubility in organic solutions [94,95]. In addition, HIPC is characterized by an increased membrane permeability, but it does not provide sufficient protection from enzymatic degradation (1-Figure 4). Introduction of HIPC to SEEDS system increases stability for enzymes, provides acceptable membrane permeability, and finally effective drug adsorption (2-Figure 4).

For instance, Gallarate et al. applied HIP technique to leuprolide and insulin to study their encapsulation in solid lipid nanoparticles (SLN). In both cases, counter-ion exchange (to docusate for leuprolide, and dodecylsulphate for insulin) made their encapsulation manageable. Moreover, they observed pH-dependent ion pair stoichiometry for leuprolide with a 1:1 ion pair of leuprolide to docusate at neutral pH and the 1:2 ratio at pH of 4. Furthermore, the ion pair formed under acidic conditions was characterized by an increased encapsulation efficiency as compared to that obtained at neutral pH. This phenomenon was related to the difference in lipophilicity of the studied ion pairs. Finally, hydrophobic ion pair-based leuprolide SLN proved to act as a sustained release system in vitro [96]. Another application of the HIP technique to improve peptide incorporation into drug carriers was reported by Karamanidou et al. In their study, a novel SEDDS for oral insulin delivery was developed. In this research, a uniform mixture of oil, surfactants, and solubilizers spontaneously formed an *o*/*w* nanoemulsion after diluting with gastrointestinal fluids. Successful incorporation of insulin into SEDDS was achieved through exchanging its counter-ion to dimyristoyl phosphatidylglycerol. Moreover, the formulation increased the enzymatic stability and mucus permeability of insulin [97]. Similar observations were made by Hintzen et al. who indicated an increase in enzymatic stability and membrane permeability of leuprolide after exchanging its counter-ion to oleate and incorporating the complex into SEDDS formulation. Furthermore, in vivo study on rats revealed an improved oral bioavailability of leuprolide oleate SEDDS as compared to that of leuprolide acetate [98]. Another SEDDS-oriented study, with desmopressin used as an active ingredient, was presented by Zupančič et al. In their study, to select the most appropriate counter-ion, different molar ratios of octadecyl sulphate, dodecyl sulfate, oleate, stearate, and docusate were assessed for their complexation efficiency. The complexation efficiency was expressed as a percent of precipitated desmopressin and was most successful for dodecyl sulphate, oleate, and docusate counter-ions. Additionally, in all cases, a specific molar ratio exhibiting maximum efficiency was determined. Both, too low (due to low complex lipophilicity) and too high (due to complex solubilization by micellization) counter-ion:peptide molar ratios resulted in inferior complex precipitation. Moreover, the influence of the aforementioned counter-ions on desmopressin lipophilicity, expressed as Capmul 907 P/water partition coefficient, was evaluated. The most efficient increase in desmopressin lipophilicity was achieved for docusate, and just this counter-ion was chosen for SEDDS formulation development. As in previous studies, peptide complexation with hydrophobic counter-ion enabled its incorporation into SEDDS and, subsequently, increased enzymatic stability [99]. The research subject was further investigated by Griesser et al. and Hetényi et al. They studied complex formation between various ratios of docusate, dodecylsulphate, and oleate counter-ions and leuprolide, insulin, and desmopressin with regard to complex formation efficiency, its lipophilicity, solubility in different solvents, and SEDDS applicability. Overall, similar to the previous article, docusate counter-ion exhibited the excellent HIP properties and, thus it was chosen as a peptide-complexing agent in SEDDS formulations. In addition, in each case, the highest quantity of the formed complexes was obtained when docusate molar amount was close to the net positive charge of peptide. The number of hydrophobic counter-ions per molecule, however, was not directly influencing lipophilicity of the complex. As the degree of lipophilicity increase depended also on peptide size, the authors suggested a number of immobilized hydrophobic counter-ions per kDa of the peptide as one of the key parameters of lipidation. In the case of each peptide, docusate counter-ion complexes proved to be efficiently loaded into SEDDS, which, in turn, increased enzymatic stability of peptides and provided their sustained release [100,101]. Lu et al. screened 15 counter-ions of different pK_a_ (acid form; ranging from −1.9 to 5.0) and logP (ranging from −4.92 to 7.60) values concerning their hydrophobic counter-ion—polymyxin B complex formation efficiency. The efficiency was based on (I) formation of precipitate in water; (II) dissolution of precipitate in tetrahydrofuran (THF); (III) reprecipitation of the complex after diluting of THF solution with water. Generally, complex formation efficiency was prominent for counter-ions characterized by higher molecular weights, higher logP values, and lower pK_a_ values of their acidic form. The authors emphasized that both the hydrophobicity and strong ionic interactions were mandatory for efficient hydrophobic counter-ion–peptide complex formation. As a matter of fact, four most promising counter-ions, dodecyl sulphate, dodecyl-benzenesulphonate, pamoate, and oleate, were chosen for further studies on nanoparticle-based formulations of polymyxin B. The properties of the nanoparticles, i.e., size, stability and release rates depended on counter-ion–molecule and counter-ion–peptide molar ratios. Specifically, nanoparticles based on dodecyl sulfate-peptide complexes were characterized by the uniform release of polymyxin B for 7 days, while those based on oleate-peptide complexes were characterized by a rapid release on the first day, followed by suppression on subsequent days [102]. The summary of Peptide Hydrophobic Ion Pairing application in lipid-based nanocarrier systems is presented in Table 3.

## 4. Counter-Ion Exchange and Measurement

### 4.1. Analytical Techniques for Counter-Ions Determination/Quantification

In the pharmaceutical industry, R&D and academic laboratories, hard evidence of purity, identity, and batch-to-batch consistency of all obtained chemicals are crucial parameters to be provided. This also applies to synthetic peptides. As part of quality control, several factors such as identity, purity, peptide concentration or mass fraction, residual solvents (e.g., water), and counter-ions should be determined [103]. Often disregarded characteristics of synthetic peptides are the type of counter-ions and their content. In most laboratories, determination and quantification of ions is performed by chromatographic separations (high-performance liquid chromatography or ion chromatography—IC), electromigration techniques (capillary electrophoresis—CE or isotachophoresis—ITP) or microtitration methods with potentiometric or voltametric detection (MT) [104,105,106]. The IC is a technique of the first choice for counter-ion determination [107]. This approach is relatively inexpensive, sensitive, and provides quantitative and qualitative data. It is widely used in the pharmaceutical industry, R&D and academic laboratories. IC is basically a chromatographic technique utilizing cationic (SCX) or anionic (SAX) exchange resins as stationary phases and buffers as mobile phases. The detection step is mostly performed by conductivity measurement. A major drawback of such approach is the high conductivity of the mobile phase. To overcome this problem, contemporary IC systems are equipped with suppressing devices that minimize the influence of mobile phase, thus increasing sensitivity and selectivity. For this purpose, two types of suppressors are used, a continuously regenerated membrane and intermittently regenerated packed-bed suppressors. Both can be electrochemically or chemically regenerated [108]. It should be noted that SPPS using Fmoc/t-but approach and followed by purification by RP-HPLC lead to peptides as TFA salts. Furthermore, TFA may occur in two forms, either directly bonded with basic residues of peptides or adsorbed in lyophilizate as a contaminant. In fact, procedures reported in the literature are usually focused on the determination of anions such as TFA, chlorides, acetates, sulphonates, and others [109]. Another approach used for both qualitative and quantitate measurements of ions is CE, based on electrophoretic migration of ionic species in the electric field. In this technique, separation is based due to different migration speeds in a capillary. Detection of simple ions that do not absorb UV radiation is achieved by indirect mode. Moreover, chromophores added to separation buffers provide a high background UV adsorption and eluting ions are recorded as negative peeks [110]. This technique was successfully adapted for counter-ions (chlorides, acetates, sulphonates, and trifluoroacetates) quantification with excellent linear response and low detection and quantification limits (LOD and LOQ) of 0.07 and 0.29 µmol/mL, respectively [111]. This versatile technique is also applicable for quantification of cations [112]. Despite many advantages including low sample consumption, low cost of chemicals and columns and fast analysis, CE of inorganic compounds has certain drawbacks. First of all, the indirect mode of detection CE has higher LOD thus being less sensitive. Limits of detection for CE are 10^−7^–10^−5^ mol/L while those for IC are 10^−7^–10^−9^ mol/L. Secondly, this method has a lower precision and reproducibility than IC [113]. Furthermore, some improvements were made to increase the sensitivity, such as sample preconcentration in the electrophoretic system or the use of capacitively coupled contactless conductivity detection (C4D) [114]. Other methods enable only a partial characterization of the counter-ions in peptide samples. For example, TFA during its removal is monitored by IR spectroscopy [115]. TFA displays a strong absorption band (1700 cm^−1^) overlapping I amide band of peptides and proteins. In addition, two other characteristic bands can be distinguished: at 1147 and 1200 cm^−1^. They enable the detection of TFA and can be used as markers during TFA^−^ exchange, for example, to chloride anions. Moreover, similar experiments can be done using nuclear magnetic resonance (NMR), where TFA^−^ can be determined using ^19^F NMR experiments based on a characteristic singlet at −75 ppm. Other anions can be determined by ^1^H NMR but this approach is limited to detect organic counter-ions only e.g., acetates. Unfortunately, this technique is useless for detection inorganic anions such as chlorides, bromides, and sulphonates [65]. Another technique reported recently utilizes monitoring of counter-ions (e.g., acetates) and peptides in a single run. This approach is based on the LC MS/MS analysis using a standard C18 column. To ionize acetate ions and enable their mass detection constant, post column infusion of ammonia solution is used. During analysis, the polarity of MS detector was switched to a positive mode allowing to perform MS/MS analysis of a peptide. This approach is especially useful during R&D and manufacture of peptide drugs since it enables to determine the level of acetates and peptides in a single run [116]. Other HPLC-based techniques are also applicable in the detection of ions, but their application highly depends on column type. According to United States Pharmacopeia (USP) and European Pharmacopeia (EP), acetate anions in peptide drugs are regarded as impurities and their levels should be measured before clinical use. One of the recommended methods for the determination of both peptides (e.g., oxytocin) and acetates is HPLC analysis using a C18 column with UV detection (220 nm) [117]. Modification of this approach is the use of a mixed-mode column, C18 with a weak anion exchange resin (WAX), enabling a faster and more precise determination of acetates in peptide drugs [118]. NP-HPLC on a monolithic silica column with evaporative light-scattering detection (ELSD) was successfully applied for the detection of cationic species, Li^+^, Na^+^, and K^+^ [119].

### 4.2. Methods of Counter-Ions Exchange

To date, several different methods of counter-ions exchange procedures were reported. However, the majority of them were based on the exchange of anions, especially trifluoroacetates to other more suitable ones. TFA anions are often exchanged to chlorides due to higher biocompatibility of the latter one. It is often accomplished by multiple dissolution of a peptide in a solution of HCl and subsequent lyophilization. Such an approach is very simple but has several drawbacks. First of all, this process is time-consuming as it needs to be repeated several times to provide a satisfactory level of exchange. Furthermore, to remove the excess of HCl another dissolution in water and freeze drying has to be performed. Secondly this method does not provide a complete TFA removal and exchange to chlorides and traces of these anions can be still recorded. In the majority of approaches, the reported efficacy reaches up to 98% after several repetitions. Thirdly, the exchange and elimination of TFA anions are limited to the stronger acids (pK_a_ of TFA = 0.52). For instance, hydrochloric acid meets this criterion with pK_a_ of −8.0 but some of the used counter-ions like acetic acid are too weak (pK_a_ = 4.76) [120,121]. Finally, this approach can lead to peptide degradation. The major degradation pathway is the hydrolysis of the peptide bond so the HCl concertation should be adjusted (lowered) in some cases [66]. Recently, our group reported a modification of this method, which utilizes the use of HCl saturated organic solvents, namely acetonitrile or *tert*-butanol. This conception is quite novel, but it proved to be highly efficient after single repetition and significantly lowered time of operation. To date, no degradation reactions were observed for TFA^−^ exchange by this approach [122]. Another useful technique utilizes a preparative HPLC. Since purification of peptides using RP-HPLC involves the use of TFA as a mobile phase modifier and during cleavage from the resin, cationic peptides are obtained as TFA salts. Despite this, it is possible to change mobile phase modifier during purification of the peptide. As a result, different forms of salts e.g., acetates are obtained. However, this approach has many drawbacks, including low efficiency and limitation of compatible counter-ions. Due to the risk of corrosion of stainless steel used in HPLC pumps, detectors and injection valves construction HCl is not suitable. Finally, this should be emphasized that the excess of acid from collected fractions has to be easily removed by evaporation or freeze drying [123].

A versatile method for TFA removal is based on the use of ion-resins modified with desired counter-ion. The resins are mostly modified with quaternary ammonium moieties like *N,N,N*-trimethylammonium. Counter-ions of resins can be simply modified by flushing them with solutions containing desired anions. In fact, two versions of exchange procedure can be applied such as addition of resin to peptide solution or passing of peptide solution through column packed with resin. In the first method, resin is filtrated but in latter one fractions eluted from column are collected. This method is very universal and provides very high exchange rates [65]. Another modification of this method includes the use of columns with resin containing carbonate anions. This allows removing anions from the peptide. Specifically, during the exchange, carbonate ions react with TFA giving CO_2_ as a product and TFA anions are captured by resin. Subsequently, the peptide is eluted from the column and collected [124]. Of course, there are more methods worth mentioning described in the literature, but they are less frequently used. Among them, dissolution of peptide sample in trifluoroethanol (TFE), evaporation of TFA under nitrogen stream [125], deprotonation/reprotonation procedure using NaOH [65], or dialysis can be distinguished [19].

## 5. Conclusions

To date, the aspect of counter-ions in peptides was not summarized in a such comprehensive review. Taken into account that peptide-based drugs are an emerging group of pharmaceuticals with nearly 20 new compounds submitted to clinical trials annually [7], a need to discuss this important issue is demanded. One of the characteristics of peptides which is the presence of basic and acidic groups determines their interactions and ability to form salts. Nonetheless, during the development of new peptide therapeutics, the influence of counter-ions is rarely taken into account. This should be emphasized that selection of the counter-ion type can be essential for the stability, solubility, and pharmacokinetic profile of final formulations. Moreover, the type and level of counter-ions can affect structure i.e., conformation and physicochemical properties of peptides. Several techniques applied for counter-ion exchange have been acknowledged. For instance, lyophilization of the solution of the desired counter-ion, evaporation from organic solvents, and the use of ion exchange resins or chromatography. Except for the counter-ion exchange itself, the ability for accurate determination of the counter-ions type and the quantity of all ions cannot be overestimated. For such purpose, a variety of techniques can be applied like ion chromatography, capillary electrophoresis, titration, and spectroscopic techniques (IR, NMR). During the development of new compounds, especially those intended for medical applications it is essential to take into account all components of final formulations. Undoubtedly, the counter-ions could be essential for expected biological and physicochemical properties.

## Figures and Tables

**Table 1:** List of selected FDA approved peptides, their sequence, type of counter-ion used, classification, and target [75,79,80,81].

Name	Sequence	Counter-Ion	Chain Length	Brand Names	Company	Route of Administration	Target/Application
Eptifibatide	*c*(Mpa-hRGDWPC)NH2	Acetate	8	INTEGRILIN^®^	Schering-Plough/Essex	Intravenous injection	Lutropin-choriogonadotropic hormone receptor, follicle-stimulating hormone receptor
Leuprolide	PHWSYLLR-NH2	Acetate	8	Eligard^®^, Enantone^®^, Lupron^®^, Memryte^®^	Atrix Labs, Takeda, Abott, Curaxis	Subcutaneous injection	Analogue of gonadotropin releasing hormone (GnRH)
Desmopressin	c(Mpa-YFQNC)PrG-NH2	Acetate	8	Nocdurna^®^	Ferring Pharmaceuticals, Inc.	Sublingual tablets	Agonist of vasopressin V1a, V1b V2 receptors
Vasopressin	*c*(CYFQNC)PRG-NH2	Acetate	9	Pitressin^®^	JHP Pharmaceuticals	Intramuscular, subcutaneous injection	Agonist of vasopressin V1a, V1b V2 receptors
Oxytocin	*c*(CYIQNC)PRG-NH2	Acetate	9	Pitocin^®^	JHP Pharmaceuticals	Intravenous infusion	Agonist of interferon alpha/beta receptor 1 and 2
Buserelin	pEHWSYs(tBu)LRP-NHEt	Acetate	9	Suprecur^®^	Sanofi-Aventis	Subcutaneous injection	Agonist of lutropin-choriogonadotropic hormone receptor and GnRH receptor
Abarelix	Ac-d-2Nal-d-4-chloroPhe-d -3-(3′ -pyridyl)AS-N(Me)YLK(iPr)Pa-NH2	Acetate	10	PlenaxisT^®^	Praecis Pharms	Intramuscular injection	Palliative treatment of men with advanced prostate cancer. GnRH antagonist that reduces the serum testosterone.
Cetrorelix	Ac-d-2Nal-d-4-chloroPhe-d-3-(3’ -pyridyl)Ala-SY-d-Cit-LRPa-NH2	Acetate	10	Cetrotide^®^	Merck Serono	Subcutaneous injection	GnRH antagonistic activity. It competes with natural GnRH for binding to membrane receptors on pituitary cells and thus controls the release of LH and FSH in a dose-dependent manner
Goserelin	pEHWSYs(tBu)LRP-NHNHCONH2	Acetate	10	ZOLADEX^®^	AstraZeneca	Subcutaneus administration	GnRH agonist for the management of locally confined carcinoma of the prostate or palliative treatment of advanced carcinoma
Histrelin	pEHWSYh(1-Bn)LRP-NHEt	Acetate	10	Vantas^®^	Endo Pharmaceuticals	Subcutaneus administration	LH-RH agonist, acts as a potent inhibitor of gonadotropin secretion, implant consists of a 50-mg histrelin acetate drug core inside a nonbiodegradable, 3 cm by 3.5 mm cylindrically shaped hydrogel reservoir
Icatibant	rRP-Hyp-G-Thi-S-d-Tic-Oic-R	Acetate	10	Firazyr^®^	Jerini AG	Subcutaneous injection	Treatment of hereditary angioedema
Triporelin	pEHWSYwLRPG-NH2	Pamoate	10	Trelstar Depot^®^	Debio Recherche Pharmaceutique	Intramuscular injection	GnRH agonist that causes a transient increase in serum testosterone levels. As a result, isolated cases of worsening of signs and symptoms of prostate cancer during the first weeks of treatment
Gramicidin D	XGALAVVVWLWLWLWY X–V or I Y–W, F or Y	Chloride	16	Neosporin^®^/Sofradex^®^	Pfizer/Sanofi	External use only, occular use	Short term treatment of steroid responsive conditions of the eye when prophylactic antibiotic treatment is also required; Otitis externa
Bivalirudin	fPRPGGGGNGDFEEIPEEYL	Trifluoroacetate	20	Angiomax^®^/Angiox^®^	The Medicines Company UK	Intravenous infusion/injection	Prothrombin inhibitor
Lucinactant	KLLLLKLLLLKLLLKLLLLK	Acetate	21	Surfaxin^®^	Discovery Laboratories, Inc.	Intratracheal administration	Lung function improvement, pulmonary surfactant
Cosyntropin	SYSMEHFRWGKPVGKKRRPVKVYP	Acetate	24	Cortrosyn^®^	Amphastar Pharmaceuticals	Intravenous injection, intravenous infusion, intramuscular injection	Agonist of adrenocorticotropic hormone receptor
Secretin	HSDGTFTSELSRLRDSARLQRLLQGLV	Acetate	27	SecreFlo^®^, Secremax^®^	Repligen Corp	Intravenous infusion	Agonist of secretin receptor
Thymalfasin	SDAAVDTSSEITTKDLKEKKEVVEEAEN	Acetate	28	Zadaxin^®^	SciClone Pharmaceuticals (SCLN)	Subcutaneous injection	Synthetic analogue of thymosin-alpha-1 for the treatment of malignant melanoma
Glucagon recombinant	HSQGTFTSDYSKYLDSRRAQDFVQWLMNT	Chloride	29	GlucaGen^®^/Glucagon^®^	Novo Nordisk/Eli Lilly	Subcutaneous, intramuscular, or intravenous infusion	Agonist of glucagon, glucagon-like peptide 1, and glucagon-like peptide 2 receptors
Sermorelin	YADAIFTNSYRKVLGQLSARKLLQDIMSRQ	Acetate	30	Sermorelin acetate^®^	Emd serono inc	Subcutaneous injection	Agonist of growth hormone-releasing hormone receptor
Nesiritide	SPKMVQGSGCFGRKMDRISSSSGLGCKVLRRH	Acetate	32	NATRECOR^®^	Scios unit of Johnson and Johnson,	Intravenous injection	Recombinant form of the B-type natriuretic peptide
Liraglutide	HAEGTFTSDVSSYLEGQAAKEEFIIAWLVKGRG	Acetate	33	Saxenda^®^, Victoza^®^	Novo Nordisk	Subcutaneous injection	Agonist of glucagon-like peptide 1 receptor
Enfuvirtide	Ac-YTSLIHSLIEESQNQQEKNEQELLELDKWASLWNWF-NH2	Acetate	36	FUZEON^®^	Roche	Subcutaneous injection	HIV fusion inhibitor, antiretroviral drug used in combinational therapy in the treatment of HIV-1
Pramlintide	K*c*(CNTATC)ATQRLANFLVHSSNNFGPILPPTNVGSNTY-NH2	Acetate	37	Symlin^®^	AstraZeneca	Subcutaneous injection	Calcitonin receptor, receptor activity-modifying protein 1, receptor activity-modifying protein 2, receptor activity-modifying protein 3
Acthar	SYSMEHFRWGKPVGKKRRPVKVYPDGAEDQLAEAFPLEF	Acetate	39	H.P. Acthar^®^	Questcor Pharmaceutical Inc.	Intramuscular or subcutaneous injection,	Adrenocorticotropic hormone (ACTH) analogue indicated as monotherapy for the treatment of infantile spasms in infants and children under 2 years of age
Corticorelin	SQEPPISLDLTFHLLREVLEMTKADQLAQQAHSNRKLLDIA-NH2,	Trifluoroacetate	41	Acthrel^®^	Ferring Pharmaceuticals, Inc.	Intravenous injection	Analogue of the human CRH (hCRH) peptide. Stimulates ACTH release and further cortisol production
Tesamorelin	X-YADAIFTNSYRKVLGQLSARKLLQDIMSRQQGESNQERGARARL-NH2 X-trans-3-hexenoic acid	Acetate	44	Egrifta^®^	Theratechnologies	Subcutaneous injection	Agonist of growth hormone-releasing hormone receptor
Aprotinin	RPDFCLEPPYTGPCKARIIRYFYNAKAGLCQTFVYGGCRAKRNNFKSAEDCMRTCGGA	Acetate	58	Trasylol^®^	Bayer Pharmaceuticals	Intravenous administration	Broad spectrum protease inhibitor which modulates systemic inflammatory response
Lepirudin	LXYTDC(1)TESGQNLC(1)LC(2)EGSNVC(3)GQGNKC(2)ILGSDGEKNQC(3)VTGEGTPKPQSHNDGDFEEIPEEYLQ X–V or T Dissulfide bridges 1-1; 2-2 and 3-3	Acetate	65	Refludan^®^	Berlex Labs	Intravenous infusion	Thrombin inhibitor, analogue of hirudin, used as anticoagulant
Mecasermin	GPETLCGAELVDALQFVCGDRGFYFNKPTGYGSSSRRAPQTGIVDECCFRSCDLRRLEMYCAPLKPAKSA	Acetate	70	Increlex^®^	Tercica, Inc.	Subcutaneous Injection	Human insulin-like growth factor-1 (rhIGF-1, rDNA origin)

*c*—cyclization through dissulfide bridge.

**Table 2:** Counter-ion influence on peptide formulations.

Peptide	Counter-Ion Exchange	Effect on Formulation Properties	Dosage Form	Ref.
Octreotide	Acetate ↓ Chloride	Slower octreotide release rate and enhanced stability	Liquid crystalline formulations	[87]
TH 9507	Acetate/Chloride ↓ Acetate + chloride	Diminished tendency towards fibril formation	Various (e.g., liquid formulation)	[89]
Teverelix	Acetate ↓ Trifluoroacetate	Elimination of gel formation	Microcrystalline aqueous suspension	[90]
Somatostatin Agonists(e.g., Lanreotide)	Acetate ↓ Oleate	Enhanced transdermal bioavailability	Transdermal patches, ointments	[91]
Exendin-4	Acetate ↓ Salicylate	Enhanced oral mucosal bioavailability	Liquid nonaqueous formulation	[92]
Leuprolide	Acetate ↓ Decanasulphonate	Enhanced pulmonary bioavailability	Aerosol formulation	[93]

Arrow (↓)—represent ion-exchange direction.

**Table 3:** The summary of Peptide Hydrophobic Ion Pairing application in lipid-based nanocarrier systems; where applicable.

Peptides	Counter-Ions	Dosage Form	Ref.
Leuprolide Insulin	Docusate (leuprolide) Dodecylsulphate (insulin)	SLN	[96]
Insulin	Dimyristoyl phosphatidylglycerol	SEDDS	[97]
Leuprolide	Oleate	SEDDS	[98]
Desmopressin	**Docusate** Dodecyl sulphate Octadecyl sulphate Oleate Stearate	SEDDS	[99]
Leuprolide Insulin Desmopressin	**Docusate** Dodecyl sulphate Oleate	SEDDS	[100,101]
Polymixin B	(1*R*)-(−)-10-camphorsulphonate 1,2-ethanesulphonate 1-decanesulphonate 1-heptanesulphonate 1-octanesulphonate 2-naphthalenesulphonate Benzenesulphonate Decanoate Deoxycholate **Dodecyl benzensulphonate** **Dodecyl sulphate** Hexanoate Myristate **Oleate** **Pamoate**	PCL/PEG-based nanoparticles	[102]

The most HIP-efficient counter-ions are in bold.

## Abbreviations

(*N*-Me)	methylated peptide bond
ACE	angiotensin-converting-enzyme
ACN	acetonitrile
AcOH	acetic acid
ACTH	adrenocorticotropic hormone
API	active pharmaceutical ingredient
ATP	adenosine triphosphate
Aβ	β-amyloid
C4D	capacitively coupled contactless conductivity detection
C_55_-P	undecaprenyl-phosphate
CCD	countercurrent distribution
CDA	calcium-dependent antibiotic
CE	capillary electrophoresis
CRF	corticotropin-releasing factor
CTAB	cetyltrimethylammonium bromide
d-2Nal	3-(2-naphthyl)-d-alanine
d-3-(3′-pyridyl)Ala	3-(3′-pyridyl)-d-alanine
d-4-chloroPhe	4-chloro-d-phenylalanine
DAAA	diamylammonium acetate
DBAA	dibutylamonium acetate
d-Cit	d-citrulline
DHAA	dihexylammonium acetate
DPAA	dipropylammonium acetate
DPC	dodecylphosphocholine
ELSD	evaporative light scattering detection
ESI-MS	electrospray ionization mass spectrometry
FA	formic acid
FDA	Food and Drug Administration
FSH	follicle-stimulating hormone
GnRH	gonadotropin releasing hormone
h(1-Bn)	1-benzyl-d-histidin
HaCaT	immortalized human keratinocytes
hCRH	human corticotropin-releasing hormone
HFBA	heptafluorobutyric acid
HILIC	hydrophilic interaction chromatography
HIP	hydrophobic ion pairing
HIPC	hydrophobic ion paired complex
HIV	human immunodeficiency virus
hR	homoarginine
Hyp	hydroxyproline
IC	ion chromatography
ITP	isotachophoresis
K(iPr)	*N*^6^-isopropyl-l-lysine
LC-MS	liquid chromatography–mass spectrometry
LH-RH	luteinizing hormone-releasing hormone
LL-37	human cathelicidin
LOD	limit of detection
MBC	minimal bactericidal concentrations
MIC	minimal inhibitory concentrations
MOG	myelin oligodendrocyte glycoprotein
Mpa	3-mercaptopropionic acid
MS/MS	tandem mass spectrometry
MSI-78	pexiganan
MT	micro titration
NaOS	1-octanesulfonate
NFPA	nonafluoropenatnoic acid
NMR	nuclear magnetic resonance
NP-HPLC	normal-phase high-performance liquid chromatography
oCRH	ovine corticotropin-releasing hormone
Oic	(3*S*,7*S*)-octahydroindole-2-carboxylic acid
PCL	polycaprolactone
pE	pyroglutamic acid
PEG	polyethylene glycol
PFPA	pentafluoropropionic acid
*p*S	*O*-phospho-l-serine
rDNA	recombinant DNA
rhIGF-1	human insulin-like growth factor-1
RP-HPLC	reversed-phase high-performance liquid chromatography
s(tBu)	*O*-*tert*-butyl-d-serine
SAX	strong anion-exchange resins
SCX	strong cation-exchange resins
SDS	sodium dodecyl sulfate
SEDDS	self-emulsifying drug delivery systems
SLN	solid lipid nanoparticles
SPPS	solid-phase peptide synthesis
TBAHS	tetrabutylammonium hydrogen sulfate
tBu	*tert*-butyl
TFA	trifluoroacetic acid
Thi	3-(2-thienyl)-l-alanine
Tic	1,2,3,4-tetrahydroisoquinoline-3-carboxylic acid
UV	ultraviolet
WAX	weak anion exchange
